# Supplementary material for: Dry Two-Step Self-Assembly of Stable Supported Lipid Bilayers on Silicon Substrates
Source: Int J Mol Sci. 2020 Sep 17;21(18):6819. doi: 10.3390/ijms21186819 (PMC7555443; doi:10.3390/ijms21186819)
Supplement: Supplementary file 1 [file ijms-21-06819-s001.zip › ijms-932909-supplementary-revS12.pdf]

Supplementary Information.

Dry two-step self-assembly of stable supported lipid bilayers on silicon substrates.

Marcelo A. Cisternas[1,2], Francisca Palacios-Coddou[1,2], Sebastian Molina[1,2], Maria Jose Retamal[2,3], Nancy Gomez-Vierling[1,2], Nicolas Moraga[1,2], Hugo Zelada[1,2], Marco A. Soto-Arriaza[2,3], Tomas P. Corrales[4] and Ulrich G. Volkmann[*1,2]

[1] Instituto de Fisica, Pontificia Universidad Catolica de Chile, Avda. Vicuna Mackenna 4860, Santiago 7820436, Chile

[2] Centro de Investigacion en Nanotecnologia y Materiales Avanzados (CIEN-UC), Pontificia Universidad Catolica de Chile, Avda. Vicuna Mackenna 4860, Santiago 7820436, Chile

[3] Departamento de Quimica-Fisica, Facultad de Quimica y de Farmacia, Pontificia Universidad Catolica de Chile. Avda. Vicuna Mackenna 4860, Santiago 7820436, Chile

[4] Departamento de Fisica, Universidad Tecnica Federico Santa Maria, Avenida Espana 1680, Valparaíso 2390123, Chile

*Correspondence: volkmann@uc.cl; Tel.: +56-2-2354-4468

Table S1. Value of different rupture forces of curves that showed only one rupture.

**Peak 1**	
Position (nm)	Height (nN)
−0.9	0.6
−4.6	6.4
−4.8	6.6
−4.8	7.1
−1.4	0.8
−5.0	6.9
−4.9	6.6
−5.4	6.7
−5.0	6.6
−5.1	6.2
−3.1	3.9
Average	5.3
Standard deviation	2.4

Table S2. Value of different rupture forces of curves that showed two ruptures.

Peak 1		Peak 2	
Position (nm)	Height (nN)	Position (nm)	Height (nN)
−0.7	0.8	−1.3	1.1
−6.6	7.0	−11.3	7.0
−4.7	6.0	−9.5	5.0
−5.0	6.9	−9.7	5.9
−5.1	5.2	−9.4	4.9
−5.6	6.6	−8.4	4.7
−5.5	6.8	−9.6	5.5
−5,6	5.9	−9.8	5.3
−5.1	5.6	−10.6	6.5
−6.0	5.5	−9.8	4.9
−5.7	6.6	−8.7	4.0
−4.8	5.4	−9.0	5.0
−4.6	6.2	−9.3	4.9
−4.3	6.0	−7.9	4.8
−5.3	5.5	−10.0	5.5
−4.0	5.4	−8.3	3.7
−5.2	6.5	−9.8	5.5
−4.8	5.3	−8.4	3.9
−4.3	4.7	−9.4	4.7
−5.7	5.0	−10.0	5.3
−4.2	5.3	−9.4	5.3
5.1	5.4	−10.6	6.3
−4.7	5.6	−9.9	6.1
−5.1	6.9	−10.3	5.8
−4,1	4.8	−9.3	4.3
−4.4	5.3	−9.3	4.9
−5.8	6.2	−9.3	4.9
−4.0	4.2	−9.3	5.2
−4.6	6.3	−8.8	6.0
Average	5.6		5.1
Standard deviation	1.2		1.1

Table S3. Value of different rupture forces of curves that showed three ruptures.

Level 3		Level 2		Level 1	
Position (nm)	Vertical Deflection (nN)	Position (nm)	Vertical Deflection (nN)	Position (nm)	Vertical Deflection (nN)
−5.7	6.9	−9.7	7.1	−13.6	6.9
−6.1	6.9	−10.0	6.6	−14.8	6.8
−6.3	7.2	−9.7	5.9	−13.6	5.7
−4.2	5.8	−8.2	5.1	−9.9	5.4
−3.6	4.4	−5.7	6.0	−9.9	4.8
−4.3	6.1	−8.8	5.8	−9.8	5.6
−2.1	2.1	−6.6	2.3	−9.1	2.7
−2.4	2.7	−4.1	2.5	−6.4	2.4
Average	5.3		5.2		5.0
Standard deviation	2.0		1.8		1.7

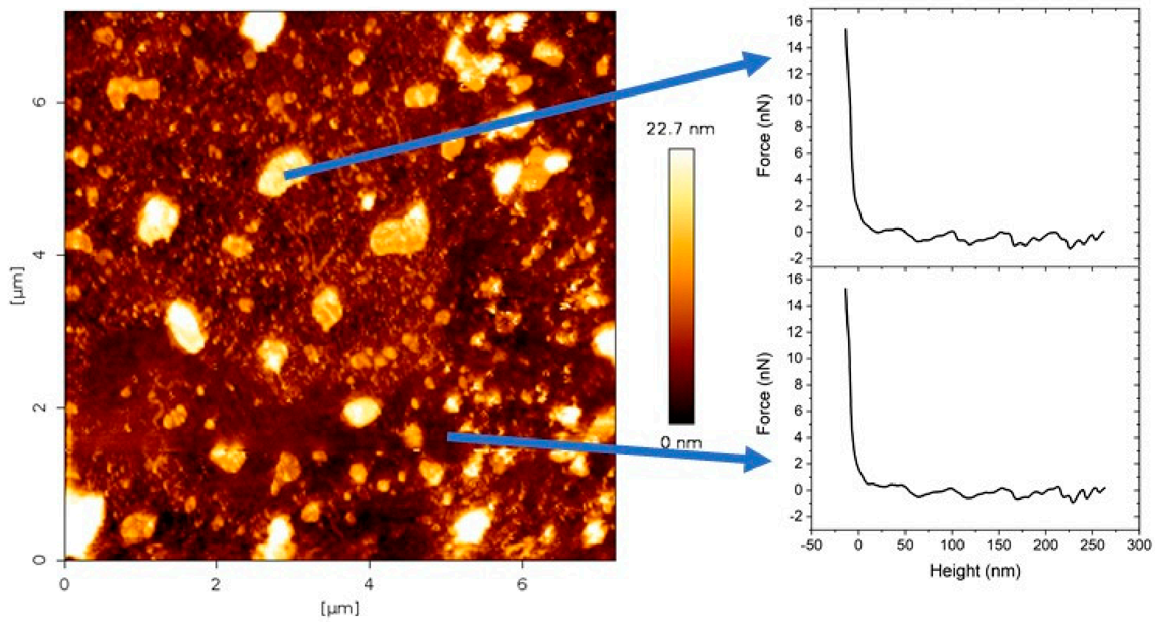

Figure S1. Left panel: Vapor deposited DPPC membrane in liquid conditions measured with AFM. Right panel: Force curves on a bilayer island (top panel) and bottom level (lower panel). Force curves show no evidence of a rupture.

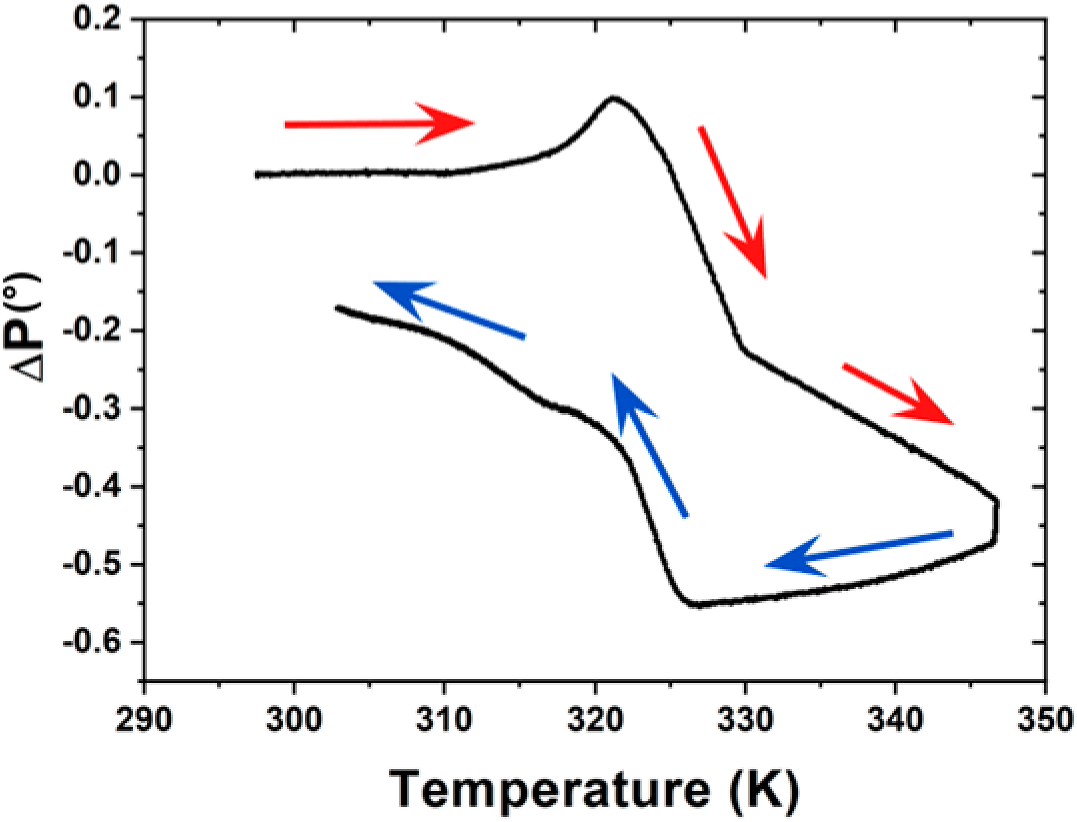

Figure S2. Temperature ramp ΔP(°) vs. T(K) corresponding to sample NE01 ramp number 7.

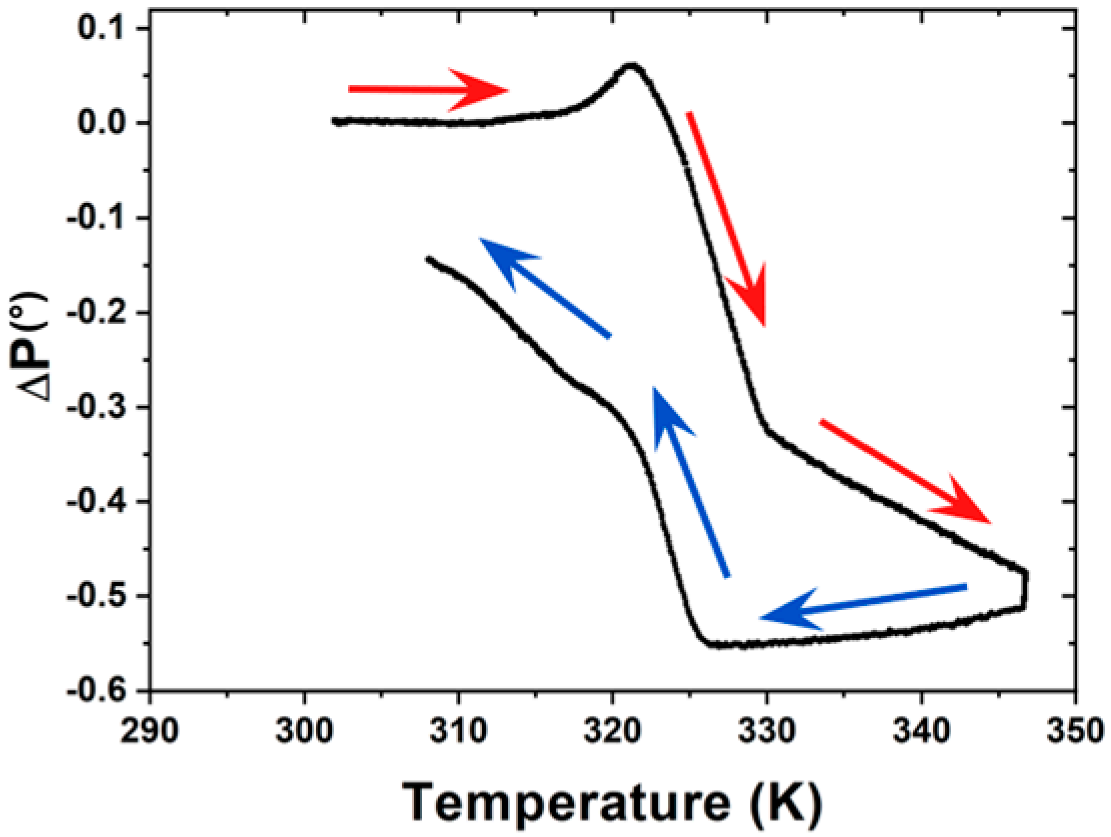

Figure S3. Temperature ramp *ΔP*(°) vs. T(K) corresponding to sample NE01 ramp number 8.

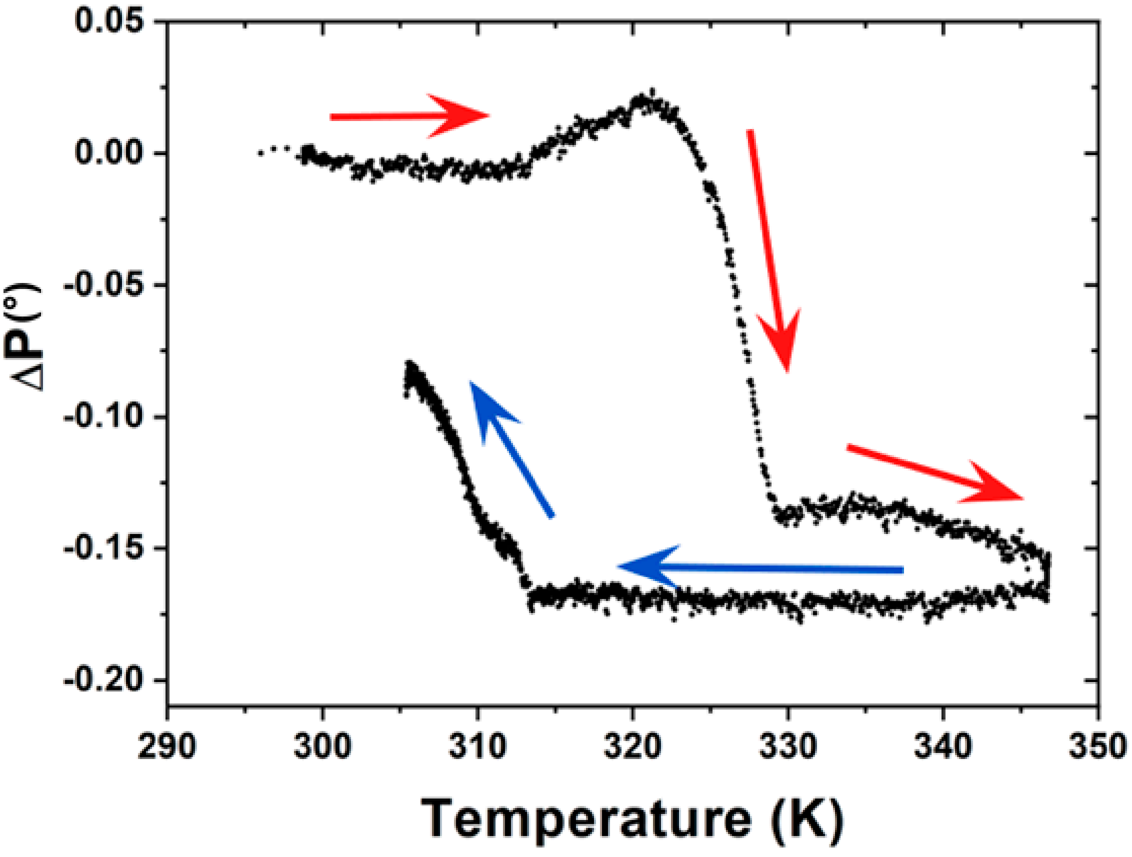

Figure S4. Temperature ramp *ΔP*(°) vs. T(K) corresponding to sample H14 ramp number 3.

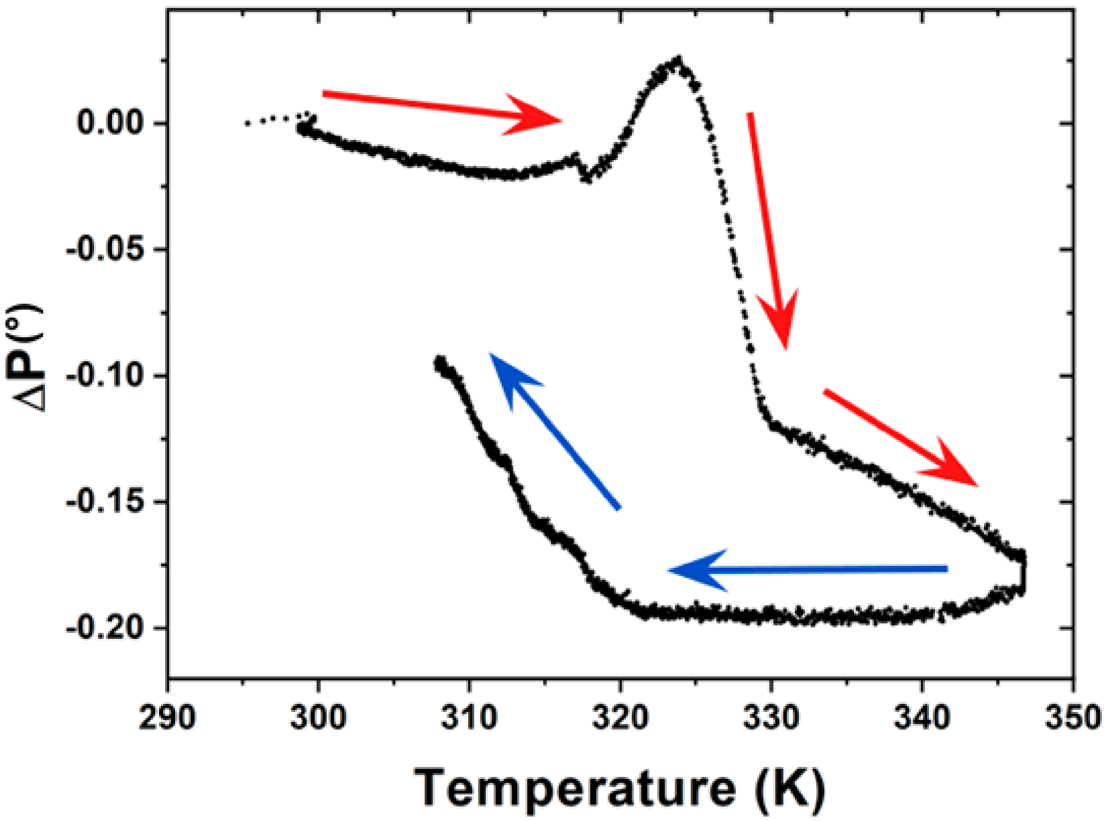

Figure S5. Temperature ramp *ΔP*(°) vs. T(K) corresponding to sample H14 ramp number 6.

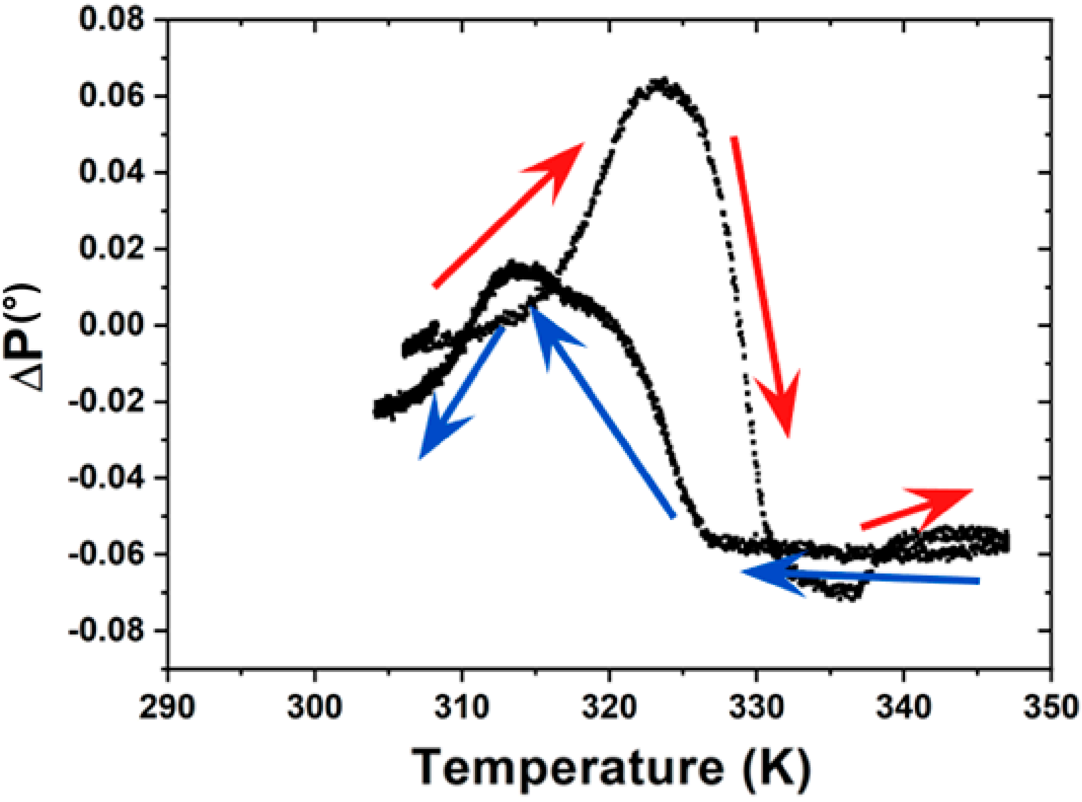

Figure S6. Temperature ramp ΔP(°) vs. T(K) corresponding to sample H15 ramp number 5.

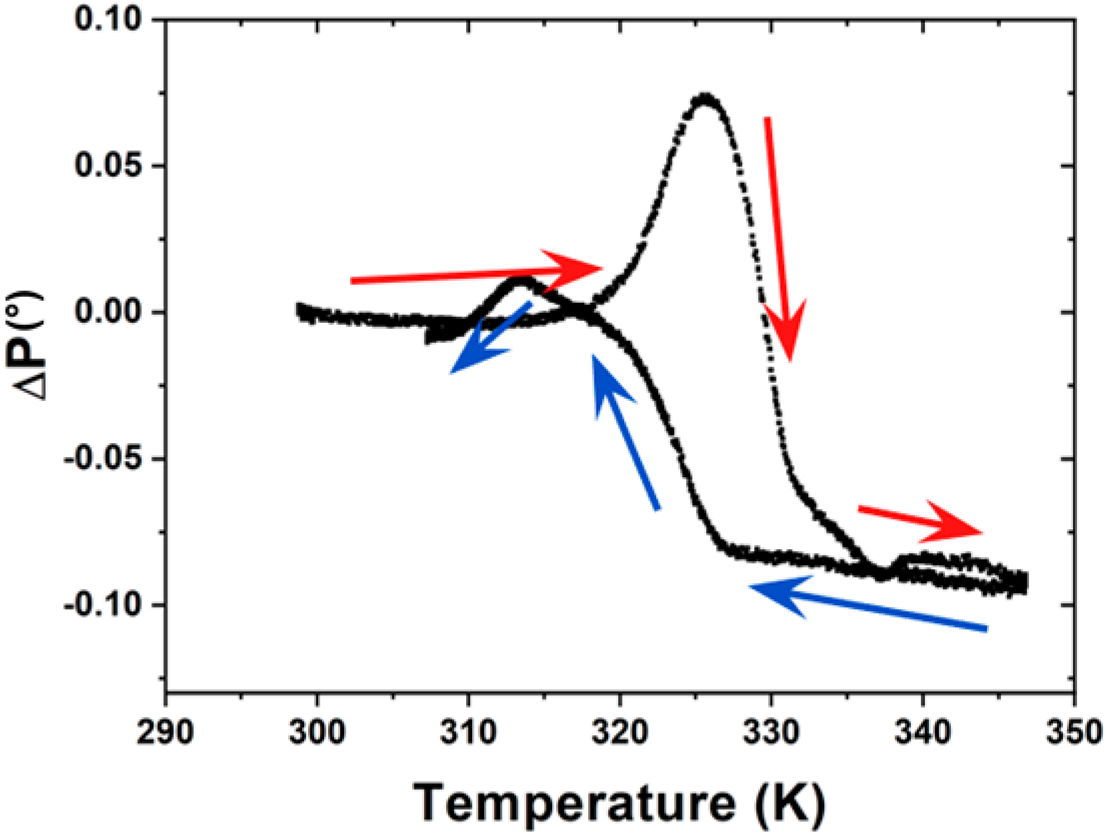

Figure S7. Temperature ramp ΔP(°) vs. T(K) corresponding to sample H15 ramp number 6.

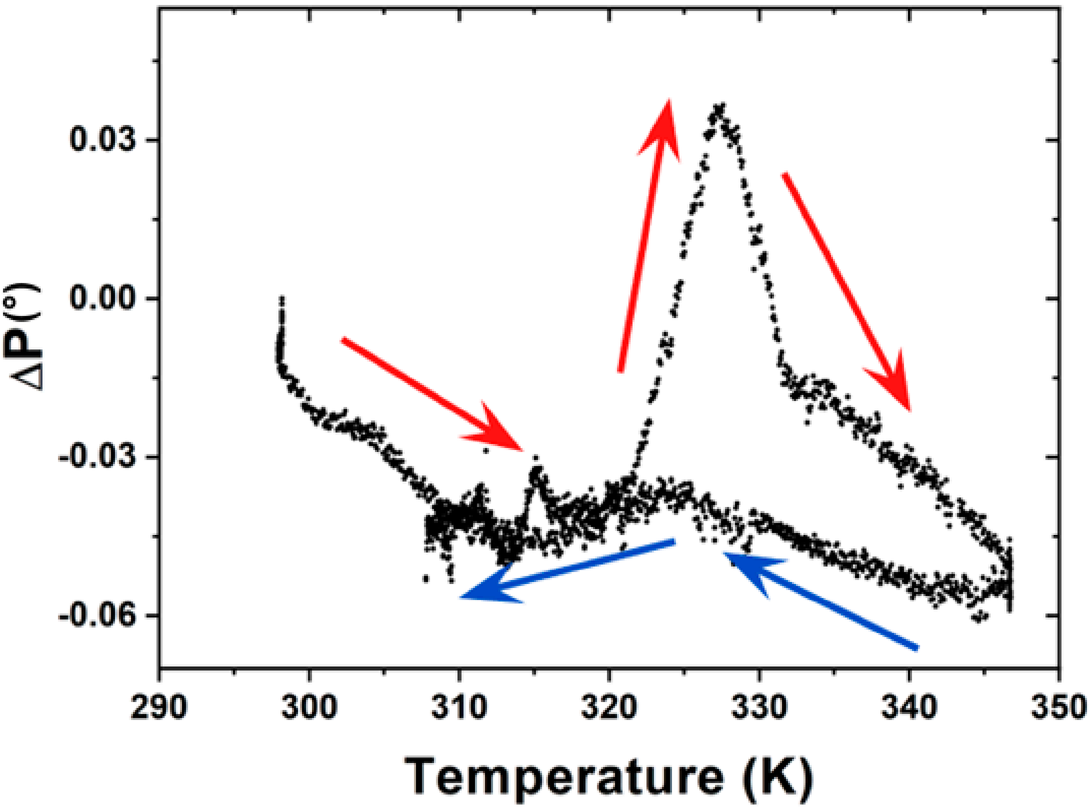

Figure S8. Temperature ramp ΔP(°) vs. T(K) corresponding to sample H16 ramp number 6.

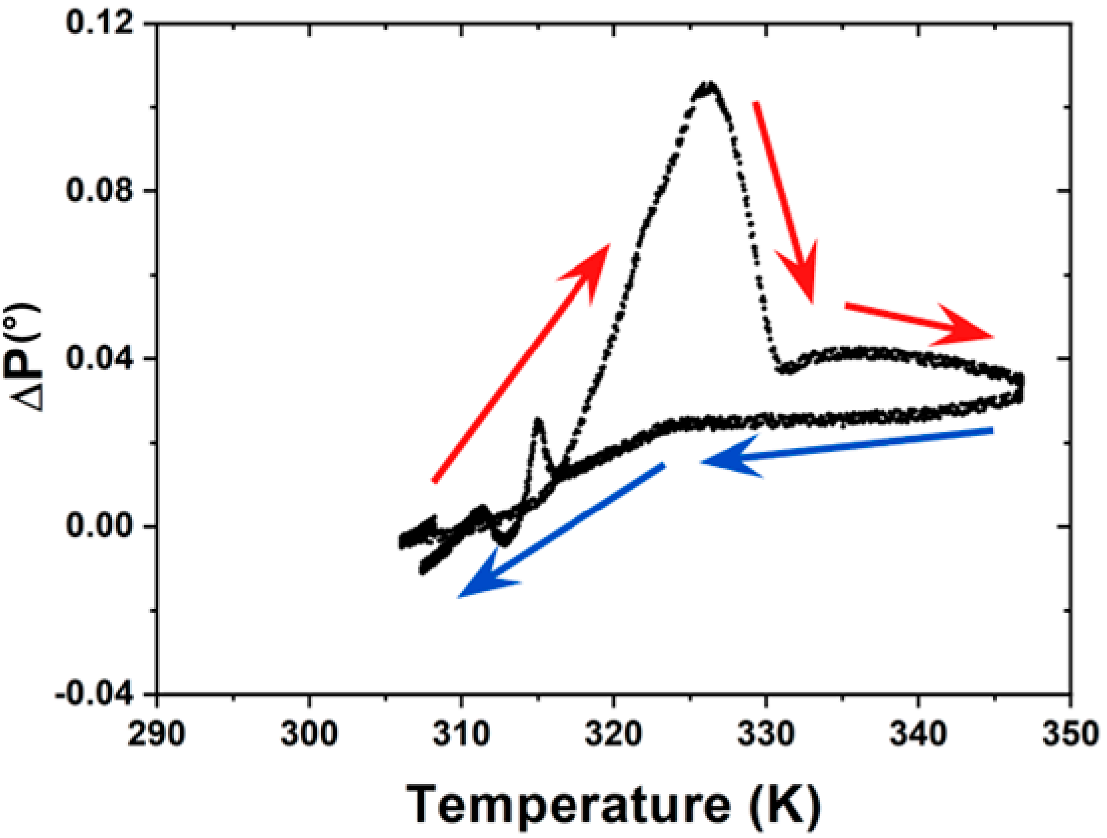

Figure S9. Temperature ramp ΔP(°) vs. T(K) corresponding to sample H16 ramp number 7.

Table S4. Values for the temperatures of three different phase transitions obtained from 48 ellipsometric measurements on 8 different samples.

Sample	Ramp number	Temp (Gel-Ripple)	Temp (Ripple-Liquid Crystalline)	Temp (LC-Fluid Disordered)
**NE01**	**7**	311.3	321.3	329.9
**NE01**	**8**	310.9	321.1	330.0
**H14**	**3**	311	321.3	329.4
**H14**	**6**	312.4	323.9	329.5
**H15**	**5**	310	323.4	330.9
**H15**	**6**	311.4	325.6	331.1
**H16**	**6**	312.8	326.6	330,3
**H16**	**7**	312.3	327.3	332.1
**Average**		**311.5**	**323.8**	**330.4**
**Standard deviation**		**0.9**	**2.5**	**0.9**

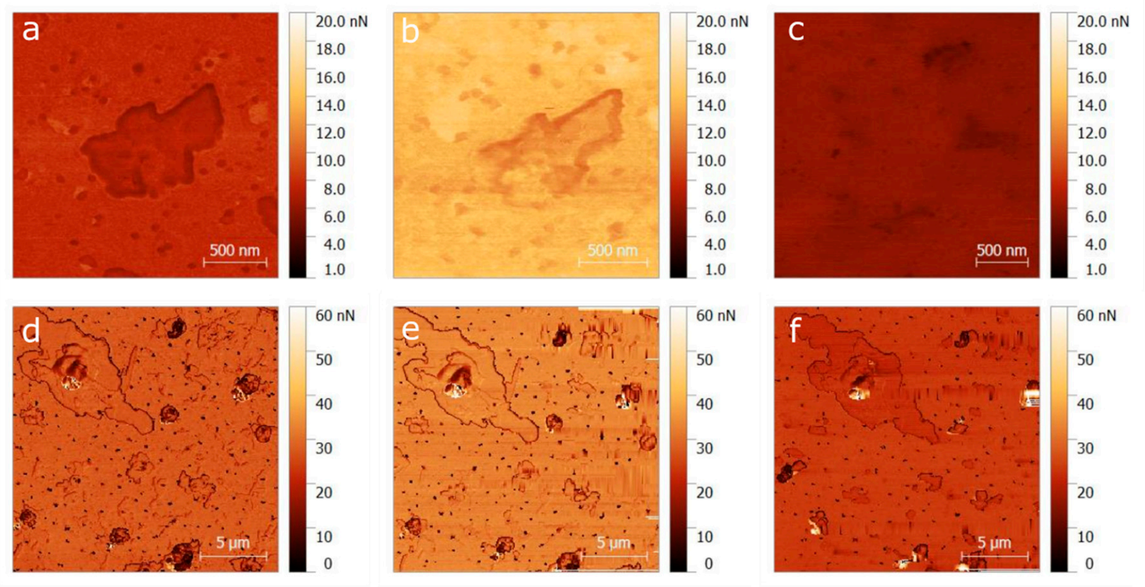

Figure S10. Adhesion maps obtained using QI™ mode for sample H14 (**a**) 296 K (**b**) 308 K and (**c**) 323 K and sample H15 (**d**) 296 K (**e**) 313 K and (**f**) 323 K.

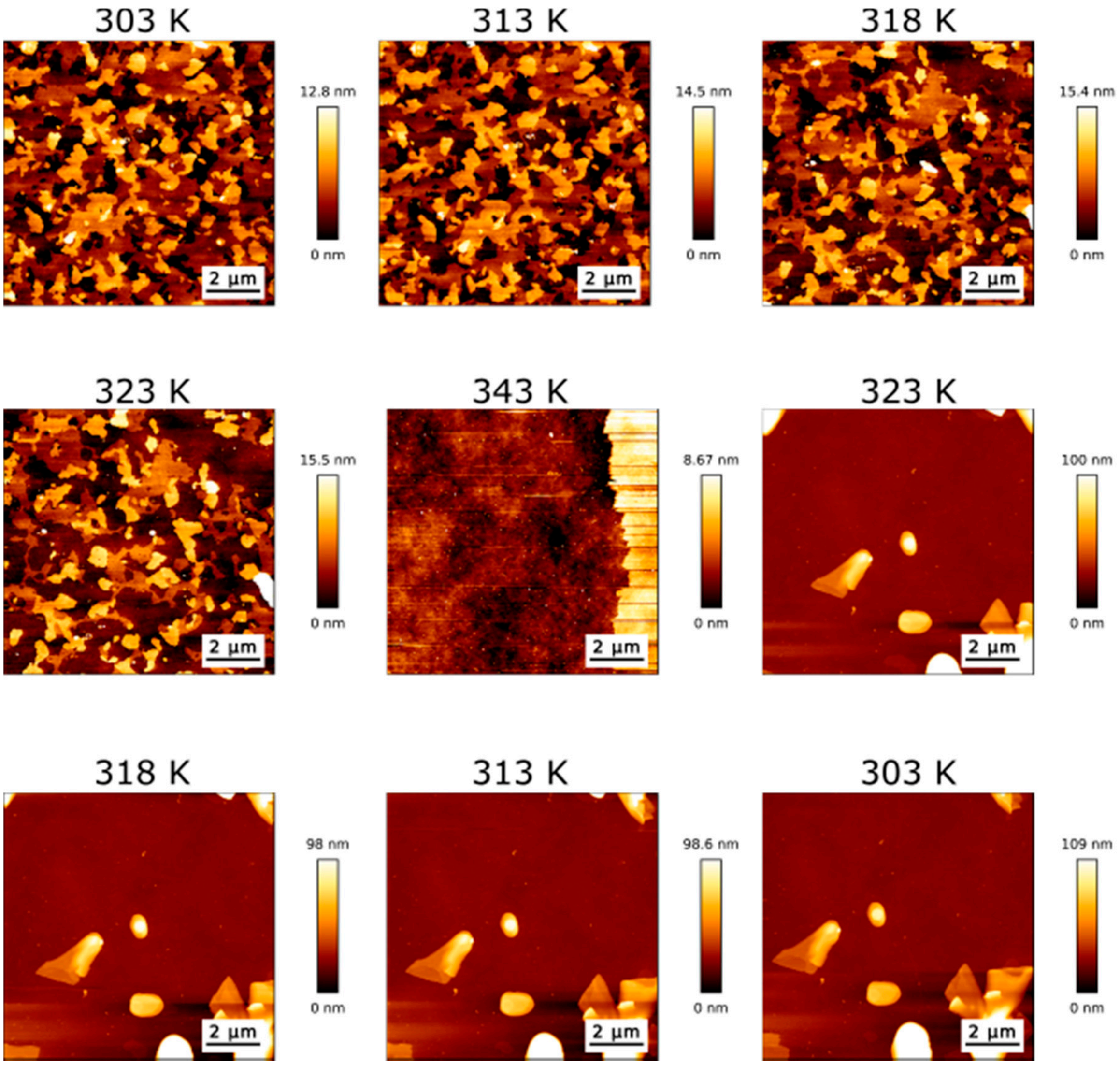

Figure S11. Topographical images of DPPC bilayer during the heating-cooling temperature cycle. At 343 K the SLB is in the fluid disordered phase and the AFM cantilever accumulates material at the right side of the image.

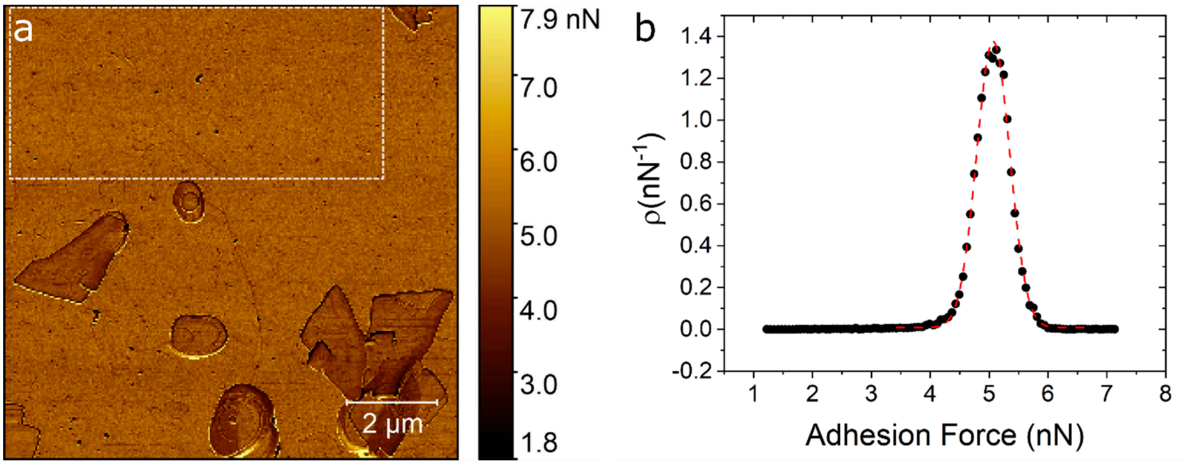

Figure S12. (**a**) Adhesion map of silicon at room temperature after a complete heating-cooling cycle corresponding to image figure 5 (e). In the marked area (white frame) the AFM-tip removed the SLB material on top of the SiO_2/Si substrate. (**b**) Histogram of the white rectangle shown in the adhesion map that represents the substrate, silicon. The main adhesion peak of silicon is around 5 nN.

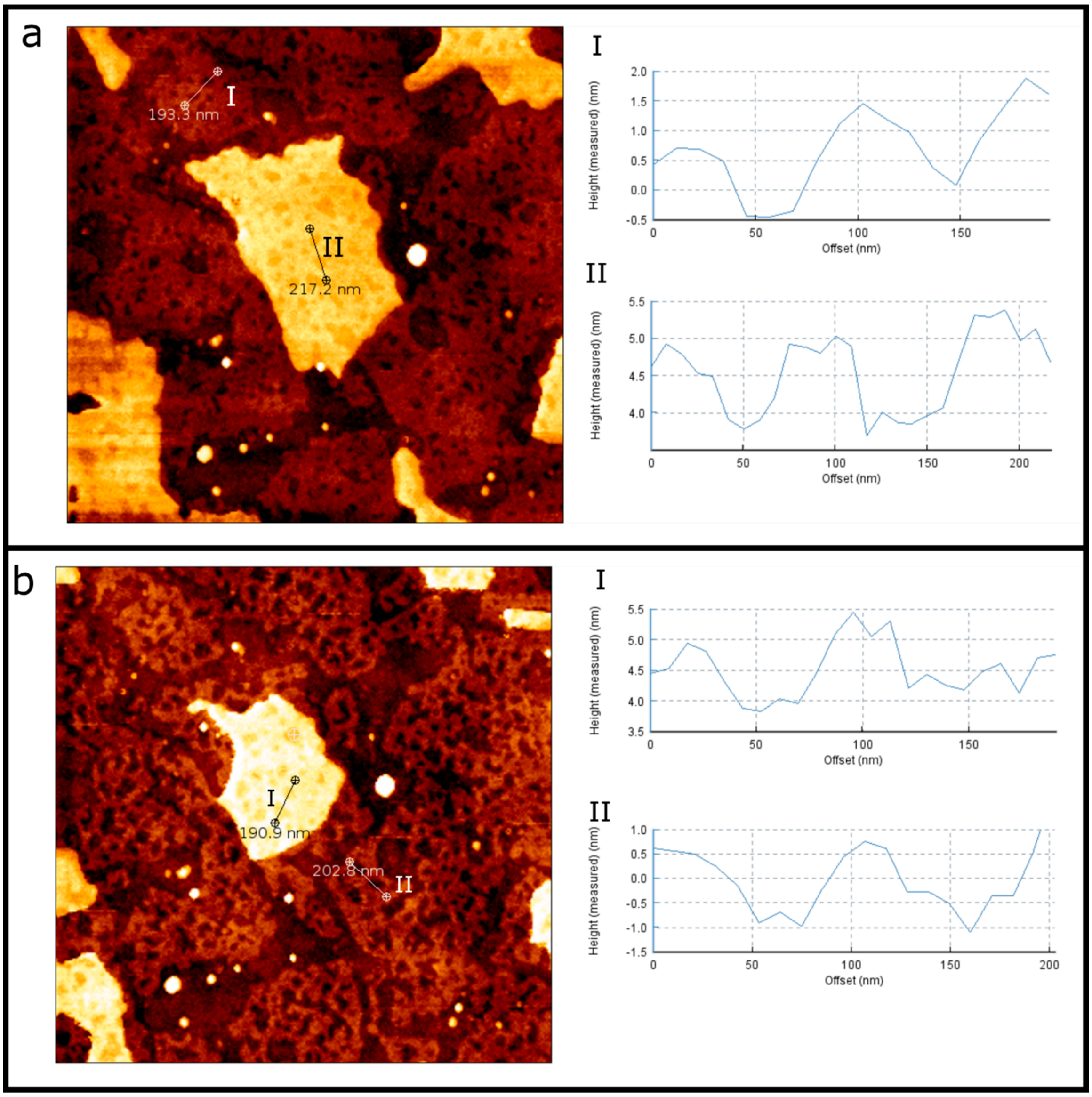

Figure S13. Topographical image and cross section of the image corresponding to (**a**) figure 6c and (**b**) figure 6f in the manuscript.

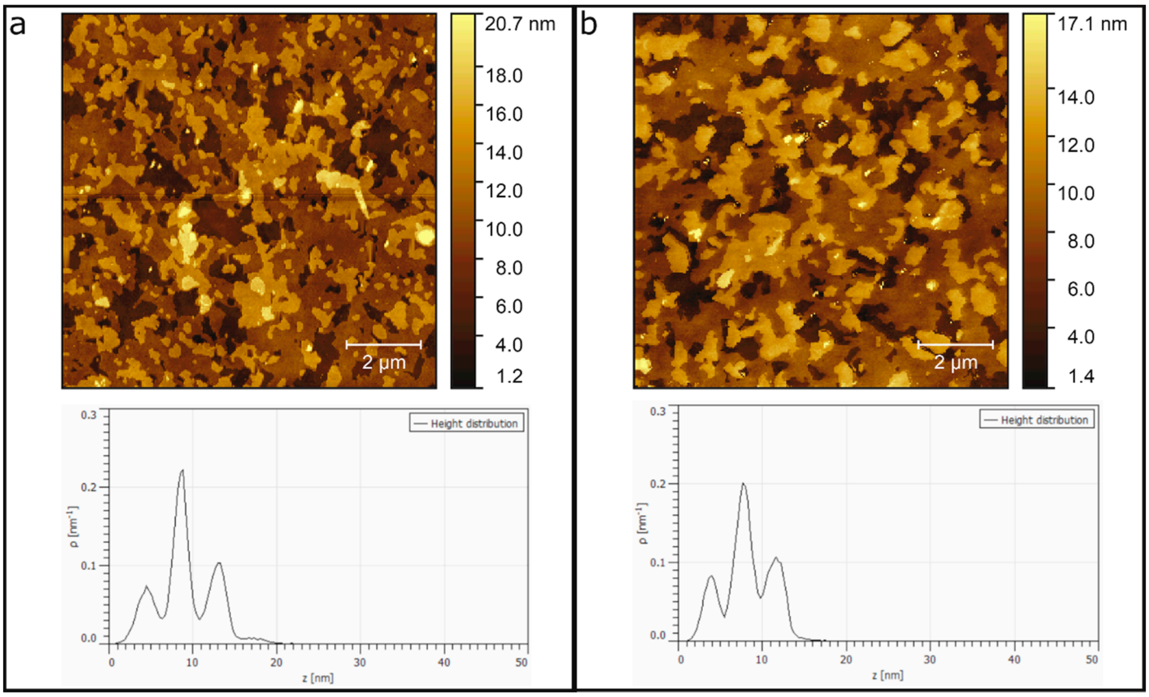

Figure S14. AFM images and histograms of (**a**) DPPC on SiO_2/Si two hours after evaporation and (**b**) nine months later. The images were taken on the same sample at different areas. The sample was stored in air inside a Petri dish in the laboratory.
